# The effect of laparoscopic ovarian drilling in patients with anovulatory polycystic ovary syndrome and high serum levels of anti-Müllerian hormone

**DOI:** 10.25122/jml-2023-0067

**Published:** 2023-07

**Authors:** Sheimaa Mohsen Mohammad

**Affiliations:** 1 Obstetrics and Gynecology Department, College of Medicine, University of Al-Qadisiyah, Al Diwaniyah, Iraq

**Keywords:** anti-Müllerian hormone, laparoscopic ovarian drilling, polycystic ovary syndrome

## Abstract

Female infertility is often associated with anovulatory polycystic ovary syndrome (PCOS), characterized by high serum levels of anti-Müllerian hormone (AMH). Laparoscopic ovarian drilling (LOD) is commonly used to treat PCOS, especially when drug interventions have failed. This study aimed to evaluate the response to LOD intervention in women with PCOS by assessing AMH serum levels and their ability to restore normal physiological menstrual cycle and achieve conception. Seventy-five infertile women (24-41 years old) with body mass index (BMI) ranging from 19.6-35kg/m2 were included in the study. Among them, 57 had primary infertility, and 18 from secondary infertility, with an average duration of 8.6±4.4 years. Baseline levels of follicle-stimulating hormone (FSH), luteinizing hormone (LH), and AMH were measured, and post-LOD levels of LH and AMH were evaluated. Menstrual cycle regularity was evaluated before and after LOD. Baseline FSH serum level before LOD was 5.2-1.6IU/L. Following LOD, the serum levels of LH and AMH significantly decreased from 14.3±4.1 to 7.8±2.8 IU/L and from 13.7±5.9 to 7.7±3.9 IU/L, respectively (p<0.05). LOD significantly (p<0.05) decreased the menstrual cycles irregularity, such as oligomenorrhea and amenorrhea, from 55 (73.3%) to 22 (29.3%) women and from 2 (2.7%) to 0 (0%) women respectively. Moreover, regular menstrual cycles significantly (p<0.05) increased from 18 (24%) to 53 (70.7%) women. Importantly, 68% of LOD-treated women showed a significant increase in pregnancy rates, with 52.9%, 35.3%, and 11.8% achieving pregnancy within 3, 6, and 9 months after LOD, respectively (p<0.05). Moreover, spontaneous ovulation was observed in 7/75 (9.3%) women within 90 days after LOD, and 71.4% achieved pregnancy. These findings highlight the success of laparoscopic ovarian drilling as an intervention for PCOS, with AMH serving as a reliable test to assess the response to LOD treatment.

## INTRODUCTION

Polycystic ovary syndrome (PCOS) is a prevalent endocrine disorder affecting approximately 4-12% of women of reproductive age worldwide [1]. Insulin resistance and obesity are common features associated with the syndrome, distinguished by a mixture of clinical or biochemical hyperandrogenism, persistent anovulatory activity, and polycystic ovaries. Although PCOS has an unknown etiology, the syndrome has been shown to cluster in families, leading researchers to assume a genetic component that is both multidimensional and multigenic [2, 3].

Until recently, the anti-Müllerian hormone was primarily associated with the sexual differentiation process in males. However, it is now acknowledged as a marker for assessing ovarian reserve and estimating the number and behavior of recruitable follicles in the early stages of growth [4, 5]. In women with PCOS, circulating and intrafollicular AMH levels are roughly two to three times higher than in healthy women. Contradictory evidence suggests that the AMH overabundance in PCOS is either caused by an intrinsically elevated synthesis by granulosa cells or is associated with a rise in the total count of preantral follicles. However, additional PCOS problems, including hyperandrogenism and insulin resistance, may also contribute to elevated AMH levels. The advancement of follicles is slowed down when AMH levels are elevated [6, 7].

In women with PCOS, LOD is performed to eliminate androgen production in the ovaries and decrease androgen-to-estrogen transformation in the periphery. LOD can decrease androgen and luteinizing hormone (LH) levels while increasing follicle-stimulating hormone (FSH) levels. Restoring an appropriate hormonal microenvironment by adjusting ovarian pituitary feedback is important to correct the endocrine alterations after surgery to transform the unfavorable estrogen-to-androgen dominated intra-follicular setting [8].

Identifying factors that influence the response of women with PCOS to LOD is essential for selecting individuals likely to benefit from the intervention. Because AMH levels are relatively stable within a given menstrual cycle, they can be used as a reliable indicator of how well a patient responds to interventions [9].

The objective of this study was to assess the response of women with PCOS to laparoscopic ovarian drilling (LOD) intervention, specifically by evaluating changes in serum levels of the anti-Müllerian hormone (AMH) and the restoration of normal physiological menstrual cycles. Furthermore, the study aimed to investigate the impact of LOD on women's ability to conceive.

## MATERIAL AND METHODS

The current study was conducted in Al-Diwaniyah Province, Iraq, between February 2021 and August 2022, at the Children and Maternity Teaching Hospital, Department of Obstetrics and Gynecology. This prospective cohort study included 75 infertile women aged 24-41 years with varying body mass indexes (BMI). Among the participants, 28 had a BMI of 19.6-25 kg/m^2^, 32 had a BMI of 25-30 kg/m^2^, and 15 had a BMI of 30-35 kg/m^2^. The inclusion criteria involved women experiencing primary infertility (57 women) and secondary infertility (18 women) for an average of 8.6±4.4 years.

During the second to the fifth day of the menstrual cycle, baseline levels of follicle-stimulating hormone (FSH), luteinizing hormone (LH), and AMH were measured using a commercial enzyme-linked immunosorbent assay kit (Immunotech, Beckman-Coulter UK Ltd., High Wycombe, Buckinghamshire, United Kingdom). The same kit and measurement method was utilized for the post-LOD assessment of LH and AMH levels.

Women with AMH levels between 4 and 8 IU/L and a history of failed drug interventions or AMH levels above 8 IU/L with a long history of infertility were used as a criterion for LOD. Menstrual cycle regularity was evaluated before and after LOD.

## STATISTICAL ANALYSIS

Hormonal values were analyzed and expressed as mean ± standard error using GraphPrism software v9. Statistical analysis was performed to detect significant differences between groups, with a significance level of less than 5%.

## RESULTS

**Figure 1:**
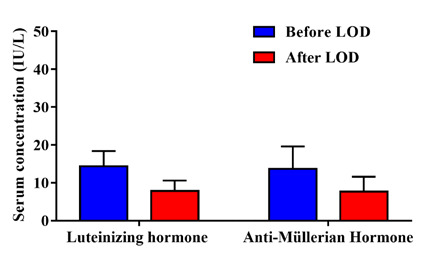
Serum concentration of luteinizing hormone and anti-Müllerian hormone in women with PCOS treated with LOD

The results showed that baseline FSH serum levels before LOD ranged from 5.2 to 1.6 IU/L. Significant reductions (p=0.0024) were observed following LOD intervention in the serum levels of LH and AMH. Specifically, the levels of LH decreased from 14.3±4.1 to 7.8±2.8 IU/L, and the levels of AMH decreased from 13.7±5.9 to 7.7±3.9 IU/L (Figure 1).

**Figure 2:**
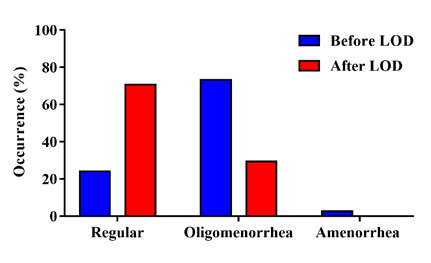
Menstrual cycle regularity women with PCOS who underwent LOD

The LOD procedure had a significant (p<0.0001) impact on reducing menstrual cycle irregularities, such as oligomenorrhea and amenorrhea. The percentage of women with oligomenorrhea decreased from 73.3% (55 women) to 29.3% (22 women), and the percentage of women with amenorrhea decreased from 2.7% (2 women) to 0% (Figure 2). Furthermore, the LOD intervention significantly (p<0.05) increased the proportion of women with regular menstrual cycles, from 24% (18 women) to 70.7% (53) (Figure 2).

**Figure 3:**
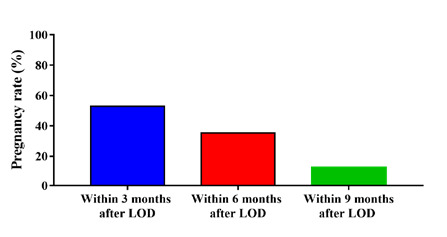
Pregnancy rate in women with PCOS who underwent LOD

The pregnancy rates among women treated with LOD were also evaluated. Among the 75 women who underwent LOD, 51 (68%) showed a significant (p<0.0001) increase in pregnancy rates. Within 3, 6, and 9 months after LOD, 27/51 (52.9%), 18/51 (35.3%), and 6/51 (11.8%) women achieved pregnancy, respectively (Figure 3). Additionally, spontaneous ovulation was observed in 7/75 (9.3%) women within 90 days after LOD, and among those, 5/7 (71.4%) successfully became pregnant.

## DISCUSSION

The current study aimed to assess the impact of laparoscopic ovarian drilling on AMH levels and the ability of women with polycystic ovary syndrome to conceive. The findings of this study are consistent with previous research by Elmashad *et al*. [10], which demonstrated a significant reduction in AMH levels after LOD therapy in individuals with PCOS [10].

Our results are also supported by a study conducted by Farzadi *et al*. [11] in Iran, where the post-LOD treatment stage showed significantly lower serum AMH levels in 30 PCOS women. The ovarian reserve was not affected by this therapy since the mean AMH was 8.4 ng/ml at baseline, 7.5ng/ml after one week, 7ng/ml after three months, and 7.7ng/ml after six months following LOD treatment. Serum AMH levels decreased by 2.13 ng/ml in PCOS females who had LOD, according to a review conducted in the United Kingdom by Amer *et al*. [12]. It is not known if this decrease was within standard ranges or whether it decreased ovarian reserve in the post-LOD treatment period. The research findings by Amer *et al*. [12] were consistent with those of the current investigation.

Research conducted by Abu Hashim *et al*. [13] in Egypt showed a decrease in serum levels of AMH following both unilateral and bilateral LOD in women with PCOS, which is consistent with the findings of our study. Additionally, an Indian study showed that following LOD for PCOS, the concentration of AMH in 30 women decreased by as much as 33% [14, 15]. Previous research indicated that a decrease in AMH levels after LOD is more indicative of a normalization phase than a dysfunctional occurrence. The number of holes drilled during LOD may influence the extent of ovarian damage, with studies suggesting that four or five holes result in milder damage [16].

Women with PCOS had elevated AMH levels, possibly linked to a higher-than-normal number of primary antral follicles. Nevertheless, some research results demonstrated that the increase in AMH concentration was not merely the consequence of a rise in the number of follicles but also attributed to an increase in AMH synthesis by each follicle. AMH is closely linked to the number of primary antral follicles and plays a crucial role in determining the size of the follicle reserve. Therefore, a decline in AMH concentrations may indicate a reduced ovarian reserve [17, 18]. The increased pregnancy rate observed in our study following LOD supports the findings reported by Paramu [19].

## CONCLUSION

This study showed that laparoscopic ovarian drilling (LOD) was an effective intervention for PCOS. The findings demonstrate a significant reduction in serum levels of AMH following LOD, indicating a positive response to the treatment. This suggests that AMH can serve as a reliable marker to assess the effectiveness of LOD in patients with PCOS.
